# Study of Sequential Dexter Energy Transfer in High Efficient Phosphorescent White Organic Light-Emitting Diodes with Single Emissive Layer

**DOI:** 10.1038/srep07009

**Published:** 2014-11-12

**Authors:** Jin Wook Kim, Seung Il You, Nam Ho Kim, Ju-An Yoon, Kok Wai Cheah, Fu Rong Zhu, Woo Young Kim

**Affiliations:** 1 Department of Green Energy & Semiconductor Engineering, Hoseo University, Asan, Korea; 2 Department of Engineering Physics, McMaster University, Hamilton, Ontario L8S 4L7, Canada; 3 Department of Physics and Institute of Advanced Materials, Hong Kong Baptist University, Hong Kong, China

## Abstract

In this study, we report our effort to realize high performance single emissive layer three color white phosphorescent organic light emitting diodes (PHOLEDs) through sequential Dexter energy transfer of blue, green and red dopants. The PHOLEDs had a structure of; ITO(1500 Å)/NPB(700 Å)/mCP:Firpic-x%:Ir(ppy)_3_-0.5%:Ir(piq)_3_-y%(300 Å)/TPBi(300 Å)/Liq(20 Å)/Al(1200 Å). The dopant concentrations of FIrpic, Ir(ppy)_3_ and Ir(piq)_3_ were adjusted and optimized to facilitate the preferred energy transfer processes attaining both the best luminous efficiency and CIE color coordinates. The presence of a deep trapping center for charge carriers in the emissive layer was confirmed by the observed red shift in electroluminescent spectra. White PHOLEDs, with phosphorescent dopant concentrations of FIrpic-8.0%:Ir(ppy)_3_-0.5%:Ir(piq)_3_-0.5% in the mCP host of the single emissive layer, had a maximum luminescence of 37,810 cd/m^2^ at 11 V and a luminous efficiency of 48.10 cd/A at 5 V with CIE color coordinates of (0.35, 0.41).

White organic light-emitting diodes (WOLEDs) have attracted significant attention for applications in flat panel displays and next generation solid-state lighting due to their advantages; surface diffusive emission, large area manufacturability, eco-friendliness and eventually cost effective fabrication process^1^,^2^,^3^. Two color and three color (blue, green and red) configurations with single and multi- emissive layers have been used for producing white phosphorescent organic light emitting diodes (PHOLEDs). Bi-directional and symmetrical illumination, semitransparent white PHOLEDs offer additional features and design freedoms for application in planar diffused lighting^4^. There are several requirements for WOLEDs used in illumination, such as; high brightness, high efficiency, high color render index (CRI), proper CIE color coordinates, high color stability and long lifetime. These requirements have been achieved through a variety of methods. The primary method is the use of phosphorescent emissive materials. PHOLEDs have electroluminescence quantum efficiencies of up to four times that of the fluorescent organic light-emitting diodes, due to the strong spin-orbital coupling induced by higher, heavy atoms^5^,^6^,^7^,^8^,^9^. There have been many methods used to achieve high luminous efficiency in WOLEDs, such as; multiple emitters in single or multi emissive layer, tandem stacks with two or three color combination, hybrid structures using both fluorescent and phosphorescent materials, and, quantum well structures^10^,^11^,^12^,^13^. In order to fabricate high efficient WOLEDs, some complicated device structures, (for example, multiple emissive units in WOLEDs), are often employed by complex fabrication processes and are faced with cost challenges in mass production^14^,^15^. However, white PHOLEDs with a single emissive layer may have a relatively limited exciton recombination zone and have the potential to meet the fabrication cost requirements due to their simple structure^16^,^17^.

In host-dopant systems, three paths can lead to phosphorescent emission by the dopant. (i) Singlet excitons formed in the host under electrical excitation can be transferred to the singlet excited state of the dopant via Foster and Dexter energy transfer processes. Then, they may be converted to triplet excitons by efficient intersystem crossing. (ii) Triplet excitons formed in the host can be transferred to the triplet excited state of a phosphorescent dopant through Dexter energy transfer. (iii) The holes and electrons can directly recombine in the dopant by charge trapping. Efficient Foster energy transfer requires the overlap of the host emission spectrum and the dopant absorption spectrum whereas efficient Dexter energy transfer requires the matching of the energy levels of the single and triplet excitons in the host with the energy levels of the excitons in the dopant. In addition, a significant offset of the HOMO and LUMO energies between the host and dopant is necessary for direct recombination in the dopant by charge trapping. In order to produce a high efficient white PHOLED with a single emissive layer, overlap between the emission spectrum of the host and the absorption spectrum of the dopant is extremely important. The Dexter energy transfer exponential reduction with distance between the host and dopant, is another important factor.

In this study, high performance three color white PHOLEDs with a single emissive layer were produced. The concentrations of three red, green and blue phosphorescent dopants were adjusted to optimize for high efficiency and CIE color coordinates and indirectly both the Foster and Dexter energy transfer processes in the host-dopant system. In order to achieve high efficiency white PHOLEDs, the overlap in the emission spectrum of mCP and the absorption spectrum of R, G, B dopants, such as FIrpic, Ir(ppy)_3_, Ir(piq)_3_, and the distance between the host and the dopants are the key factors to achieve efficient energy transfer processes and hence high luminous efficiency. Sequential energy transfer between the host and three phosphorescent dopants in the single emissive layer and the phenomenon of red shift in the electroluminescent (EL) spectra as a function of dopant concentration are discussed.

## Experimental

**Figure 1:**
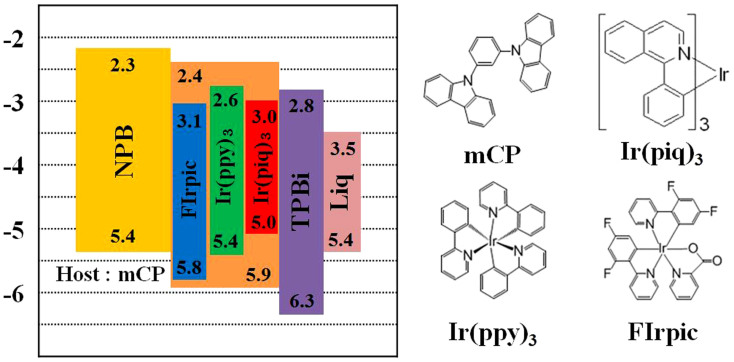
Schematic energy band diagrams of white PHOLEDs and molecular structures of host and dopant materials.

To fabricate OLED devices, Indium tin oxide (ITO) coated glass substrates, with a sheet resistance of ~12Ω/square, were used. ITO patterns were formed by photolithography processes. The pre-patterned ITO glass substrates were cleaned ultra-sonically with (sequentially) de-ionized water, isopropyl alcohol, acetone, de-ionize water, and isopropyl alcohol. This was followed by O_2_ plasma treatment under vacuum conditions of 5.0 × 10^−2^ Torr, at 50 W for 2 minutes. All organic materials were deposited by thermal evaporation under a base pressure of ~1.0 × 10^−7^ Torr. The white PHOLEDs composed of; a N,N′-diphenyl-N,N′-bis(l-naphthyl-phenyl)-(l,l′-biphenyl)-4,4′–diamine (NPB) hole transporting layer (HTL), N,N′-dicarbazolyl-3,5-benzene (mCP) host material, blue, green and red phosphorescent dopants of bis(3,5-difluoro-2-(2-pyridyl)-phenyl-(2-carboxypyridyl)iridiumIII (FIrpic), tris(2-phenylpyridine)iridium(III) (Ir(ppy)_3_), and tris(1-phenylisoquinoline)iridium(III) (Ir(piq)_3_), a 2′,2′,2″-(1,3,5-Benzinetriyl)-tris(1-phenyl-1-H-benzimidazole) (TPBi) electron transporting layer (ETL) and an 8-Hydroxyquinolinolato-lithium (Liq) electron injection layer. All organic materials were purchased from Luminescence Technology Corp. or Electronic Materials Index Co., Ltd and used as received without further purification (>99%). The aluminum cathode was deposited by thermal evaporation at a rate of 5.0Å/s. The electro-optical characteristics of the white PHOLEDs were measured using a Keithley 238 LMS PR-650 spectrophotometer, a colorimeter and an IVL system. Photo absorption and emission spectra of the host and dopant molecules were measured in CH_2_Cl_2_ at room temperature. Fig. 1 describes the energy band diagrams of white PHOLED devices. The parameters of different white PHOLEDs, fabricated with varying doping concentrations of blue, green and red phosphorescent dopants, are summarized in Table 1.

## Results and Discussion

**Figure 2:**
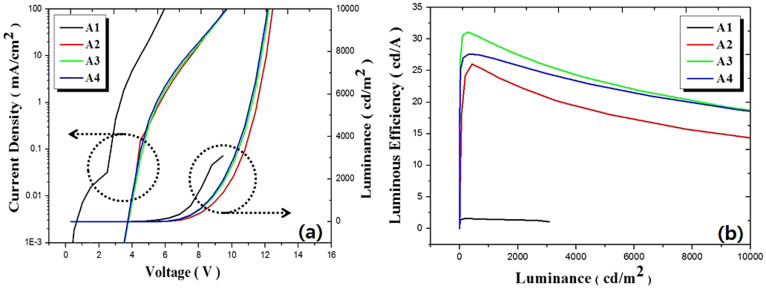
(a) Current density-voltage (J-V) and luminescence-voltage (L-V) measured for devices A1–A4, and (b) plots of luminous efficiency as a function of luminescence.

It has been reported that quenching processes due to triplet-triplet exciton annihilation (TTA) and triplet-polaron annihilation are responsible for the efficiency roll-off^18^. In this work, the dopant concentration of FIrpic used in the PHOLEDs was adjusted (to achieve high efficiency), by optimizing the EL spectra, taking into account the self-quenching and/or TTA of the dopant molecules. We fabricated devices A1–A4 by varying the doping concentration of FIrpic from 0% to 12% as described in Table 1. Fig. 2 (a) shows the current density–voltage (J–V) and luminance–voltage (L–V) measurements for devices A1–A4, while Fig. 2 (b) shows luminous efficiency as a function of luminance (~10000 cd/m^2^). As shown in Fig. 2 (a), the initial current density of devices A2–A4 increased with the FIrpic concentration. Device A4 has higher current density than that measured for devices A2 and A3 until 50 mA/cm^2^. After that, the current density curve of the devices A2, A3 and A4 are similar to each other. Charge carriers moved through the guest molecules at the initial lower voltages and then moved through both the host and guest molecules in the EML. This is due to the fact that the FIrpic dopant has a smaller energy gap, lower HOMO and LUMO levels than that of the ETL, the HTL and the mCP host as shown in Fig. 1. However, driving voltages of devices A2, A3 and A4 is higher than that of device A1, which has an undoped EML. Device A1 has a threshold voltage of 3 V, while that for devices A2 and A3 is 4 V, and it is 4.5 V for device A4. This phenomenon arises from the band gap difference in host (broader) and dopants (narrower) in the EML. Hence, the dopant molecules are function as deep trapping centers for charge carriers in the EML causing an increase in driving voltage^19^. Therefore, self-quenching and TTA by directly recombining excitons in the dopant molecules are inevitable in a host-dopant system with higher doping concentration.

As shown in Fig. 2 (b), the luminous efficiency of devices A1–A4 increased as the concentration of FIrpic increased from 0% to 12%. However, the efficiency of device A4 decreased due to self-quenching and/or TTA process in the dopant molecules, showing that the FIrpic concentration below 12 wt% is preferred^20^.

**Figure 3:**
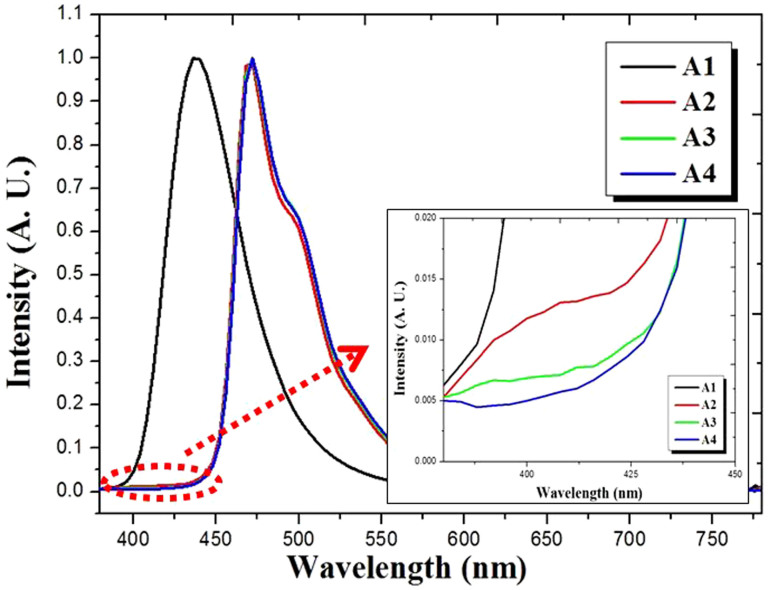
EL spectra measured for devices A1, A2, A3 and A4.

Fig. 3 shows the EL spectra measured for devices A1–A4. It provides evidence of energy transfer from the mCP host to the phosphorescent dopant FIrpic following the FIrpic doping concentration from 0% to 12%. This result shows a complete energy transfer from host to phosphorescent dopant, except in the case of device A2 (with lower doping concentration of 4%), as mCP's emission peak at 410 nm has almost disappeared. A slight emission remained at around 410 nm (see the inset of Fig. 3) in the spectra of device A2 and is caused by incomplete energy transfer from mCP to FIrpic at the low doping concentration of FIrpic. We suggest that the absence of the emission peak at around 410 nm shows that energy from excited states is rapidly transferred from the host to the dopant. For devices A3 and A4, the results indicate a complete energy transfer from mCP host to FIrpic dopant. Therefore, an 8% concentration of FIrpic was fixed and was considered as having complete energy transfer from the fluorescent host mCP to the phosphorescent dopant FIrpic with no energy loss from self-quenching or TTA processes.

**Figure 4:**
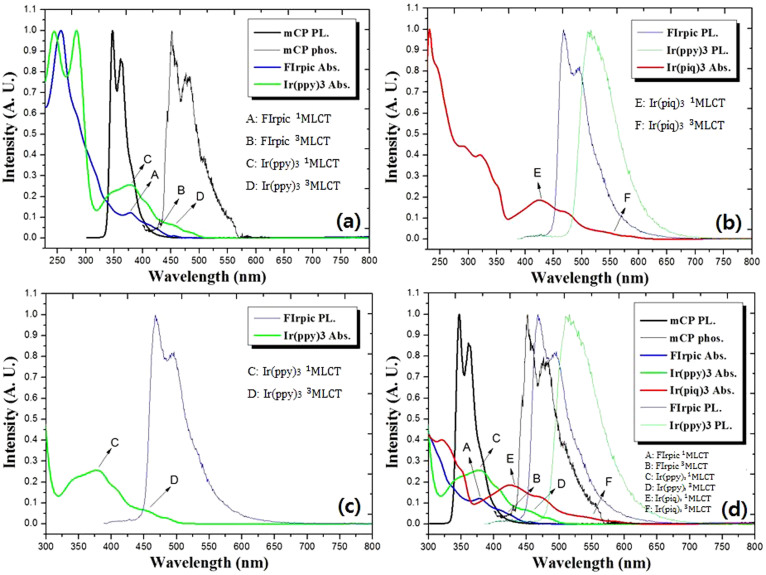
Photoluminescence and phosphorescence spectra measured for mCP, FIrpic and Ir(ppy)_3_ and the absorption spectra of FIrpic (blue), Ir(ppy)_3_ (green) and Ir(piq)_3_ (red) dopants.

**Figure 5:**
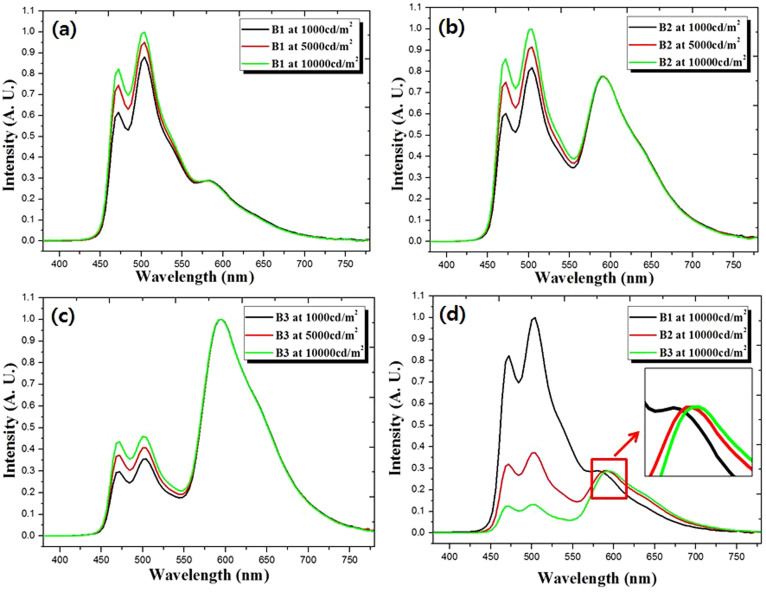
EL spectra of the devices with different concentrations of red Ir(piq)_3_ dopant.

To optimize the doping concentrations of different primary dopants (R, G and B) in a single emissive layer in white PHOLEDs, both the overlap between the photo absorption spectra of the dopants and the emission spectra of the host, and, the match of energies in the host-dopant and/or dopant-dopant system should be considered. As shown in Fig. 4, the Ir complexes exhibit an intense ligand ^1^π-π* absorption band centered around 250–350 nm with a weak absorption band extending from 350 nm into the visible region, which originats from the metal-to-charge transfer (MLCT)^17^,^21^,^22^. As shown in Fig. 4 (a), the PL/phosphorescence spectra of mCP overlaps well with the MLCT absorption bands of Ir(ppy)_3_ centered from 350 nm to 500 nm, implying an efficient Foster energy transfer from host to Ir(ppy)_3_. On the other hand, the MLCT absorption of FIrpic is centered from 350 nm to 450 nm, which means there is almost no overlap with the PL/phosphorescence spectrum of mCP above 450 nm, implying insufficient Foster energy transfer occurs from the mCP to FIrpic as compared with mCP to Ir(ppy)_3_. However, as shown in Fig. 4 (b), the intensity of blue and green emission from FIrpic and Ir(ppy)_3_ may be reduced by Foster energy transfer from both FIrpic and Ir(ppy)_3_ to Ir(piq)_3_. The spectral overlap between PL of FIrpic/Ir(ppy)_3_ and MLCT of Ir(piq)_3_ centered from 430 nm to 600 nm can be clearly recognized. It is indicated that the MLCT of Ir(piq)_3_ overlaps well with the PL of FIrpic and Ir(ppy)_3_. Also the MLCT of Ir(ppy)_3_ overlaps well with the PL of FIrpic from 440 nm to 500 nm as shown in Fig. 4 (c). The rate constant k_en_^D^ of Dexter energy transfer mechanism can be expressed^23^ by the following two equations: 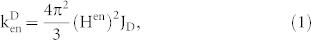

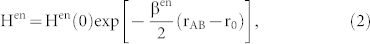
where H^en^ is the electronic term that can be obtained from the electronic coupling between donor and acceptor and is exponentially dependent on distance (β^en^ is a physical constant for energy transfer between donors and accepters), the nuclear factor J_D_ is the spectral overlap between the emission spectrum of the donor and the absorption spectrum of the acceptor. We need to consider the optimization of dopant concentrations to achieve a well-balanced three primary color white emission according to both Foster and Dexter energy transfer processes using the two equations above. As shown in Fig. 4 (b) and (c), FIrpic has a lower J_D_ than the other dopants due to a large overlap with absorption of Ir(ppy)_3_ and Ir(piq)_3_ which means Ir(ppy)_3_ and Ir(piq)_3_ molecules will absorb FIrpic's energy. Therefore, to achieve a well-balanced primary color (R, G and B) emission for proper white CIE color coordinates, the concentration of FIrpic should be higher than the concentration of Ir(ppy)_3_ and Ir(piq)_3_, rendering the distance between mCP and FIrpic closer for higher H^en^. However, despite relatively lower doping concentrations of Ir(ppy)_3_ and Ir(piq)_3_ which means they have lower H^en^ compared to the FIrpic in the EML, FIrpic has weak emission than the emission from other dopants due to Dexter energy transfer processes from higher T_1_ to lower T_1_ as shown in Fig. 5 (b) and (c). Indeed this result suggests that highly efficient blue emission by direct recombination of excitons in FIrpic is difficult to be control. There is a weak blue emission compared to the other emissions in spite the higher doping concentration of FIrpic. And highly efficient green and red emission by direct recombination of excitons in Ir(ppy)_3_ and Ir(piq)_3_ is also difficult to be control due to the extremely lower doping concentrations.

The excited triplet states (T_1_) of mCP, FIrpic, Ir(ppy)_3_ and Ir(piq)_3_ are 2.9 eV, 2.6 eV, 2.4 eV and 2.1 eV which is calculated by energy of the emission peak at PL spectrum, respectively. The green and blue emission spectra, peaked at 500 nm and 470 nm, measured for devices B1, B2 and B3 at 1000 cd/m^2^, 5000 cd/m^2^ and 10000 cd/m^2^ are plotted in Fig. 5 (a), (b) and (c). This result can be explained as a saturation process. When two or three different phosphorescent dopants are close to each other within the Dexter radius, the energy transfer process occurs from higher T_1_ to lower T_1_ until the dopant in the vicinity with the lower T_1_ are fully excited by triplet excitons.

Fig. 5 (d) shows a shift in the red emission peak of the EL spectra of white PHOLEDs as a function of Ir(piq)_3_ with increase in concentration from 0.1% to 1%, measured at 10000 cd/m^2^. This is a result of the previously mentioned phenomenon of deep trapping centers for charge carriers in the guest molecules in the EML. Device B3 appears more red shifted than the other devices, B1 and B2 a due to higher doping concentration of Ir(piq)_3_. In turn, this suggests that the amount of trapped charge carriers generated by the dopant molecules are proportional to the concentration of Ir(piq)_3_^24^,^25^.

**Figure 6:**
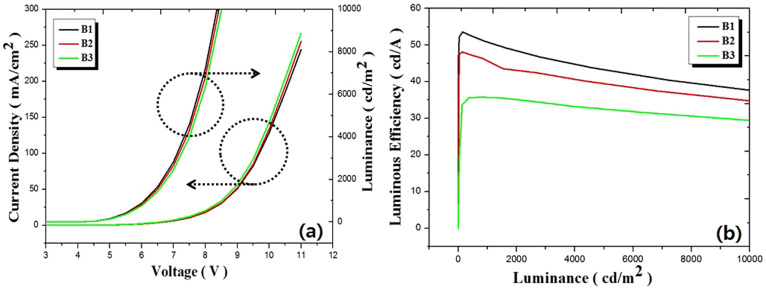
(a) Current density – voltage and luminescence – voltage characteristics and (b) luminous efficiency as a function of luminescence measured for devices B1 and B3.

Fig. 6 (a) shows the current density-voltage and luminance-voltage characteristics measured for devices B1–B3, and Fig. 6 (b) shows the luminous efficiency as a function of luminance (~10000 cd/m^2^). The devices B1, B2 and B3 showed maximum luminescence of 37290 cd/m^2^, 37810 cd/m^2^ and 38020 cd/m^2^ at 11 V, and luminous efficiency of 53.60 cd/A, 48.10 cd/A and 35.58 cd/A at 5 V with CIE color coordinates of (0.25, 0.42), (0.35, 0.41) and (0.47, 0.40), respectively. Even though the current density curve of device B1 is lower than those of the other devices, B2 and B3, the luminance curve of device B1 is higher than those of devices B2 and B3. These results indicat that Ir(piq)_3_ molecules are rapidly saturated by excitons. After Ir(piq)_3_ molecules are saturated, the excitons on Ir(piq)_3_ may lead to self-quenching. Therefore, the excitons may contribute to deterioration in luminescence as red doping concentration increases.

## Conclusion

The concentration of three dopants FIrpic, Ir(ppy)_3_ and Ir(piq)_3_ in the EML was optimized (for high efficiency and CIE color coordinates), with consideration for the spectral overlap between the emission spectrum of the donor and the absorption spectrum of the dopants. The radius of molecular the system in the Dexter energy transfer process plays an important role in achieving sufficient energy transfer. Therefore, the doping concentration of Ir(ppy)_3_ (green) and Ir(piq)_3_ (red) should be much lower than that of FIrpic due to stronger spectral overlap in the single emissive layer in white PHOLEDs. The concentration of FIrpic (blue) dopant in the EML should be higher than the others because it is related to the electronic coupling between the donor and acceptor which is exponentially dependent on distance. Using optimized phosphorescent dopant concentrations of Firpic-8.0%:Ir(ppy)_3_-0.5%:Ir(piq)_3_-0.5% in the mCP host of the single emissive layer, white PHOLEDs showed a maximum luminescence of 37,810 cd/m^2^ at 11 V, and luminous efficiency of 48.10 cd/A at 5 V, with CIE color coordinates of (0.35, 0.41). Consequently triplet excitons were properly transferred from host to dopant and/or from dopant to dopant generating a well-balanced emission without energy loss through optimizing the concentration of the three phosphorescent dopants in a single emissive layer of white PHOLED devices.

## Author Contributions

K.J.W. and K.W.Y. wrote main manuscript text and Y.S.I. provided most of I-V-L characteristics data including Fig. 2 and 6. K.N.H. and Y.J.A. provided EL spectra on Fig. 4. C.K.W. and Z.F.R. suggested valuable discussion about energy transfer mechanism describing in Fig. 4.

## Figures and Tables

**Table 1:** Summary of different dopant combinations used in the single EML of white PHOLEDs of the type: ITO(1500 Å)/NPB(700 Å)/EML(300 Å)/TPBi(300 Å)/Liq(20 Å)/Al(1200 Å)

Device	EML (300 Å)
**A1**	mCP:FIrpic-0%
**A2**	mCP:FIrpic-4%
**A3**	mCP:FIrpic-8%
**A4**	mCP:FIrpic-12%
**B1**	mCP:FIrpic-8%:Ir(ppy)_3_-0.5%:Ir(piq)_3_-0.1%
**B2**	mCP:FIrpic-8%:Ir(ppy)_3_-0.5%:Ir(piq)_3_-0.5%
**B3**	mCP:FIrpic-8%:Ir(ppy)_3_-0.5%:Ir(piq)_3_-1.0%
